# Exploring Esophageal Widening in Computed Tomography Performed in Critically Ill Patients

**DOI:** 10.3390/jcm15218638

**Published:** 2026-11-06

**Authors:** Hany Kayed, Mohamed I. Soltane, Svetlana Hetjens, Florian Rehberger, Thomas Walter, Dominik Nörenberg, Stefan O. Schoenberg, Fabian Siegel, Johann S. Rink

**Affiliations:** 1 Department of Radiology and Nuclear Medicine, University Medical Centre Mannheim, Medical Faculty Mannheim, Heidelberg University, D-68167 Mannheim, Baden-Württemberg, Germany; hany.kayed@umm.de (H.K.); dominik.noerenberg@umm.de (D.N.); stefan.schoenberg@umm.de (S.O.S.); 2 Department of Diagnostic and Interventional Neuroradiology, University Medical Centre Mannheim, Medical Faculty Mannheim, Heidelberg University, D-68167 Mannheim, Baden-Württemberg, Germany; mohamedilyes.soltane@umm.de; 3 Department of Medical Statistics and Biomathematics, University Medical Center Mannheim, Medical Faculty Mannheim, Heidelberg University, D-68167 Mannheim, Baden-Württemberg, Germany; svetlana.hetjens@umm.de; 4 Integrated Emergency Center, University Medical Centre Mannheim, Medical Faculty Mannheim, Heidelberg University, D-68167 Mannheim, Baden-Württemberg, Germany; florian.rehberger@umm.de (F.R.); thomas.walter@umm.de (T.W.); 5 Mannheim Institute for Intelligent Systems in Medicine (MIISM), Medical Faculty Mannheim, Heidelberg University, D-68167 Mannheim, Baden-Württemberg, Germany

**Keywords:** critically ill patients, esophageal widening, computed tomography

## Abstract

**Background/Objectives**: Early diagnosis and therapy stratification represent central factors in the management of critically ill patients, today often assisted by clinical scores, which also include laboratory testing results. The aim of this study was to further characterize computed tomography-detected abnormal esophageal widening, which we often observe in critically ill patients in our emergency department when reading CT examinations. We expect this factor to be potentially meaningful for clinical evaluation and prognosis of critically ill patients. **Methods**: In this retrospective observational study, critically ill patients receiving CT trauma room imaging in our hospital’s emergency department were included. In the first part of the analysis, esophageal diameter at four anatomical landmarks were compared between patients who survived and those who died within 28 days in all critically ill patients presenting with AEW. In the second exploratory part of the study, the relevance of the laboratory parameters and AEW in predicting mortality were analyzed. In part three, the correlation between the 28-day mortality of all critically ill patients and the presence of esophageal widening was assessed. **Results**: Esophageal widening was detected in 98 of all 1683 included patient examinations. Nineteen of these patients have died (19.4%), and ten of them (52.6%) suffered primarily from a cerebrovascular event. In the group of patients with AEW, cervical AEW was more frequent among deceased patients (63.2% vs. 27.8%; *p* = 0.0064), and mean maximal esophageal diameter was also greater among deceased patients (19.2 vs. 16.4 mm; *p* = 0.0392). Multiple laboratory parameters were also found to correlate with mortality in all patients with AEW. No significant correlation of AEW alone with 28-day mortality in all critically ill patients that received ED-CT could be demonstrated in our retrospective sample. **Conclusions**: Esophageal widening, especially in the upper segment, was found in a substantial proportion of critically ill patients, with increased width correlating with mortality risk in the group of patients with AEW. Future studies should include much larger patient cohorts and a prospective design to evaluate if AEW might be a candidate to serve as a potential parameter of clinical scores which aim at early patient stratification.

## 1. Introduction

Critically ill patients (CIPs) require prompt medical management in emergency departments (EDs) to reduce delays to subsequent treatment, a strategy that has been described to reduce mortality [1]. With computed tomography (CT) being increasingly available and accessible in EDs, CT imaging in many cases has become part of initial management of critically ill patients [2]. Emergency care workflows in EDs have evolved, as has the integration of ED-provided care with critical care strategies, which are supported by described decreases in in-hospital mortality [3]. Given the increasing complexity and resource demand of ED management for critically ill patients, the allocation and prioritization of scarce resources represents a major and increasing challenge [4]. Objective and early assessment of a patient’s condition, along with prediction of clinical care trajectories, are therefore essential in the management of these cases.

Critically ill patients (CIPs) typically receive CT imaging in addition to clinical and laboratory assessment upon arrival in our hospital’s ED. Established instruments that aim at early therapy stratification typically focus on subgroups of patients, like the Sequential Organ Failure Assessment (SOFA) score, which provides a standardized measure of organ dysfunction for patients presenting with sepsis, relying on both clinical and laboratory parameters [5]. Multiple specialized scores have been developed to quantify the chance of survival in patients presenting with alcoholic steatohepatitis and other liver diseases, often relying on parameters like age, serum creatinine, serum bilirubin and International Normalized Ratio (INR) [6,7,8]. Critical care scores like the APACHE II score aim at quantifying mortality risk taking into account the combination of physiological parameters collected over 24 h, age and multiple medical history parameters [9]. To guide initial management of critically ill patients in the ED in a broad population of patients, reliable predictive biomarkers which are available early in the course of patient management are crucial. As CT imaging within the ED has become increasingly relevant [10], imaging-derived biomarkers could be of additional use, if included in clinical scoring approaches.

In clinical practice, we observed abnormal esophageal widening (AEW) in a subset of CIP admitted to the ED, which stimulated further exploration of this observation. The esophagus, a circular-shaped muscular structure, may be well-assessed on CT imaging [11]. From a surgical perspective, it can be divided into 4 segments [12,13]. Its lumen may not exceed 10 mm in axial CT imaging slices [11], according to published data. The presence of intraluminal fluid, air-fluid levels, or of an air-filled lumen that exceeds 10 mm in transverse diameter can be considered abnormal [14]. Whereas widening of the esophagus can also occur in long-standing untreated achalasia, we perceived dilated esophagus as an individual finding occurring especially in critically ill patients who died. We found no published reports that evaluate the presence of esophageal widening in CIP without pre-existing achalasia.

The aim of the presented study was to further characterize CT-observed esophageal widening in critically ill patients, to correlate esophageal width with mortality, evaluate its significance in combination with established laboratory biomarkers and explore a potential association of AEW alone with 28-day mortality.

## 2. Materials and Methods

### 2.1. Study Population

This retrospective study was approved by the local ethics committee II of the Medical Faculty Mannheim, University of Heidelberg, Germany (reference number 2023-896). In this analysis, our institution’s radiology information system (RIS; Siemens Syngo Portal, Siemens Healthineers AG, Forchheim, Germany) was searched for patients who received total body CT in the ED based on clinical suspicion of critical illness. Between 1 May 2022 and 31 October 2023, all identified CT scans were analyzed for the presence of AEW by a senior radiologist (>5 years of experience in CT imaging (HK)) and a board-certified radiologist (>5 years of experience (MIS)). Additionally, demographic and laboratory data were collected and evaluated.

### 2.2. Inclusion and Exclusion Criteria

The inclusion criteria for the study applied to all male and female patients aged 18–99 years who were admitted to the ED in the aforementioned time span and who received an urgent thoracoabdominal CT scan according to the internal “emergency room” protocol upon arrival in the ED. This protocol is typically applied to all cases involving patients who are critically ill and require immediate imaging. Patients suffering from a known medical cause for AEW such as achalasia—whether it be idiopathic [15] or secondary [16]—along with patients with AEW caused by distal mechanical obstruction, neuromuscular motility disorders or systemic connective tissue diseases were excluded from the study.

### 2.3. Total Body CT Imaging Procedures

All CT examinations were performed on a 1*128-slice multidetector CT (MDCT) system (Somatom Definition AS+, Siemens Healthcare GmbH, Forchheim, Germany). The acquisition parameters used were: 120 kVp tube voltage, 139 mAs current, collimation of 0.6, pitch of 1, rotation time of 0.5 s, and a reconstructed slice thickness of 0.8 mm. A contrast agent was administered via antecubital venous access using a contrast volume of 100 ccm Iomeprol 350 (Imeron 350, Bracco Imaging S.p.A., Milan, Italy), followed by a saline flush volume of 30 ccm with an injection rate of 5 ccm/s. In all total body CT scans, imaging of neck and chest was acquired in the caudocranial direction.

### 2.4. Image Analysis and Laboratory Measurements

**Figure 1:**
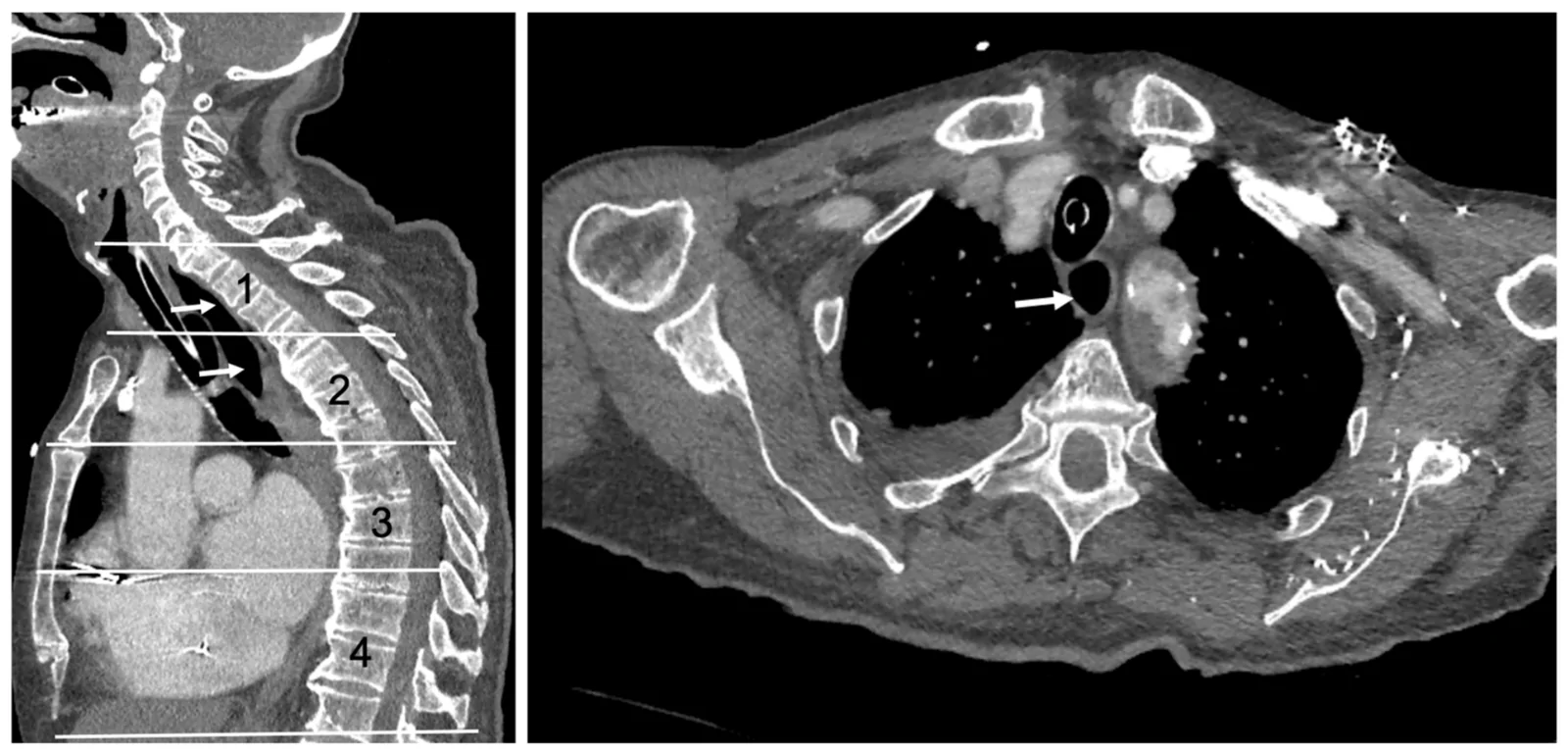
CT imaging in sagittal (**left**) and axial plane (**right**) of a CIP, which demonstrates the regional segments of the esophagus, (1) cervical, (2) upper thoracic, (3) mid-thoracic and (4) lower thoracic (numbers are placed on the thoracic spine to define the region in which the esophageal segment is located). Note the dilated esophagus (white arrows) in both images. Measurement of the esophagus lumen in millimeters was performed in axial plane on the widest part of each esophageal segment. Measured diameters > 10 mm in were defined as presence of AEW.

The esophagus was manually segmented into 4 craniocaudal anatomic segments (Figure 1) as described in the literature [12,13]. Esophageal luminal anterior–posterior diameter was measured in each of the 4 esophageal segments. The diameter measurements were performed in millimeters (mm) at the widest part of each level via axial imaging using Aycan Osirix version 3.0 (Aycan Digitalsysteme GmbH, Würzburg, Germany) by the observers. A diameter of more than 10 mm was considered an abnormal widening of the esophagus, based on the reference by Buchanan et al. [11,17]. Serum levels of creatinine and total bilirubin were analyzed from a venous blood sample from the ER. The resulting parameters were extracted from the hospital laboratory information system (LIS). All patient-level data were stored in a Microsoft Excel version 16 spreadsheet (Microsoft Corporation, Redmond, WA, USA).

### 2.5. Analysis Design

Part 1: Characteristics of esophageal widening and association of esophageal width with mortality

To characterize details on esophageal widening, all critically ill patients presenting with AEW were further analyzed. The mean axial diameter of the esophagus in all four esophageal segments were compared between the CIP who died and who survived to unravel potential correlation between esophageal widening and mortality using *t*-tests.

To understand potential bias, the analyses were repeated excluding patients who had been resuscitated or intubated before CT.

Part 2: Exploratory correlation of serum biomarkers with mortality and logistic regression analysis for prediction of death

To enable hypothesis-building, laboratory biomarkers (serum sodium, potassium, glucose, bilirubin, alkaline phosphatase, gamma GT, Aspartate Aminotransferase, Alanine Aminotransferase, lactate, C-reactive protein, NT-proBNP, creatinine, leukocyte count, Procalcitonin, Troponin I, D-dimer, hemoglobin, Albumin, Thrombocyte count) were noted in all patients presenting with esophageal widening. To confirm these parameters are suitable for predicting mortality, these were correlated with 28-day mortality using Fisher’s exact test.

Additionally, a stepwise binary logistic regression analysis was performed to identify variables associated with mortality which might serve as a potential clinical score. Death was modeled as the binary outcome (death = 1). Candidate variables included age, cervical AEW, admission category, Potassium, sodium, total bilirubin, alkaline phosphatase, lactate, creatinine, proBNP, leukocyte count, D-dimers, and hemoglobin. Variables were included into a stepwise selection procedure with a significance level of *p* < 0.05 Odds Ratios (ORs) with 95% confidence intervals (CIs) were calculated. Model discrimination was assessed using the area under the receiver operating characteristic (ROC) curve.

Part 3: Correlation of AEW alone with mortality in all critically ill patients

To explore a potential association between the presence of AEW alone and 28-day mortality in all critically ill that receive urgent CT imaging, Pearson Chi-Square and Fisher’s exact test (*p*-value, Odds Ratio, Risk Ratio/Relative Risk with 95% CI) were utilized. Mortality was additionally compared for subgroups of patients with different age groups (<50 years, 50–65 years, >65 years).

### 2.6. Statistical Analysis

Statistical analyses were performed using SAS^Ⓡ^ version 9.4 (SAS Institute Inc., Cary, NC, USA) and Microsoft Excel version 16 (Microsoft Corporation, Redmond, WA, USA). Descriptive statistics were used to describe the demographic characteristics and to analyze survival. Nominal and ordinal variables were characterized using absolute and relative frequencies. Quantitative approximately normally distributed parameters were presented as mean values and their standard deviations; for non-normally distributed data, median and ranges were provided. Normal distributions were analyzed using the location parameters and the Shapiro–Wilk test. For nominal or ordinal characteristics, the χ^2^ test or Fisher’s exact test were used accordingly. Means of two groups were compared using two-sample *t*-tests. Relevant variables were included in a multiple binary stepwise regression analysis. The area under the ROC curve (AUC) was assessed for quantifying the predictive ability of the model. *p* ≤ 0.05 was considered statistically significant.

## 3. Results

### 3.1. Baseline and Demographic Analysis

**Figure 2:**
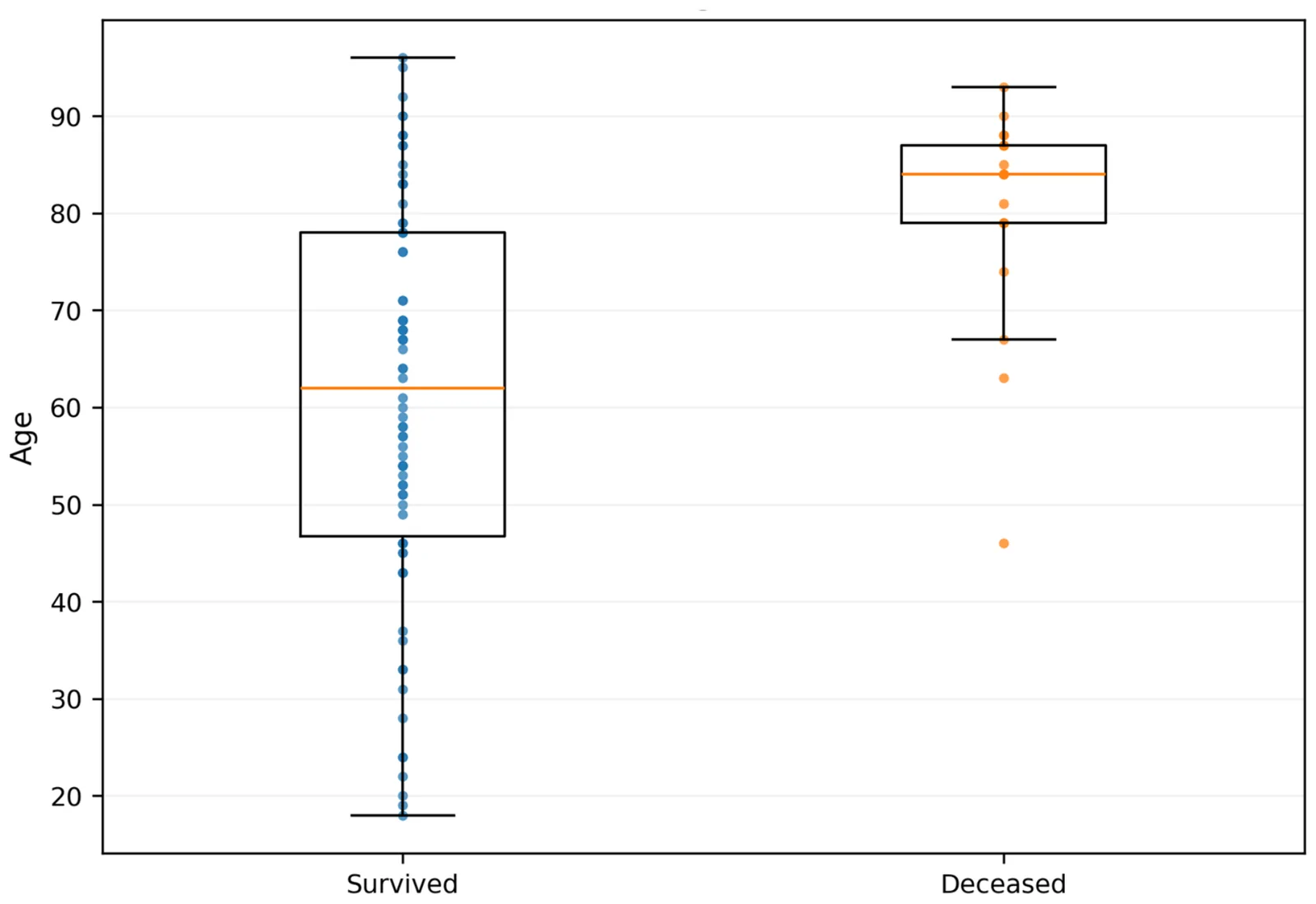
Boxplot showing the distribution of age (in years) of CIPs who survived (left) and who died (right) among AEW patients. Individual values are represented as small circles, mean (x) and median (horizontal lines); *p* = 0.0001. Blue circles: Age distribution of patients who survived, orange circles: age distribution originating from patients who died.

This study included *n* = 1683 patient examinations. *n* = 98 (5.82%) patients exhibited widened esophageal lumen (Table 1). Among those, *n* = 19 patients (19.4%) died within 28 days while *n* = 79 (80.6%) survived. The mean time of survival after CT imaging was 3.5 ± 5.3 days, with a median of 3.5 days (0–19 days). The mean age of the deceased patients was 80.3 ± 11.2 (46–93) years, while the mean age of the surviving patients was 61.2 ± 20.2 years (18–96 years) (Figure 2). This difference in age of 19.1 years was found to be statistically significant (*p* < 0.0001).

In the subgroup of AEW patients, 6/19 (31.6%) of deceased patients were females. From the surviving patients with AEW, sex information was available for 78 of the 79 survivors. Of these, 32/78 (41.0%) were female. There was no significant difference in gender found between these groups (*p* = 0.4494).

#### Reason-of-Admission Analysis

Among the *n* = 19 patients who were deceased, *n* = 10 patients suffered primarily from CVI (52.6%) and *n* = 9 patients suffered from other severe conditions (47.4%) (Table 2). Among the *n* = 79 who survived, *n* = 32 patients presented with CVI (40.5%), whereas *n* = 47 patients did not suffer from CVI (59.5%).

### 3.2. Part 1: Characteristics of Esophageal Widening and Association of Width with Mortality

**Figure 3:**
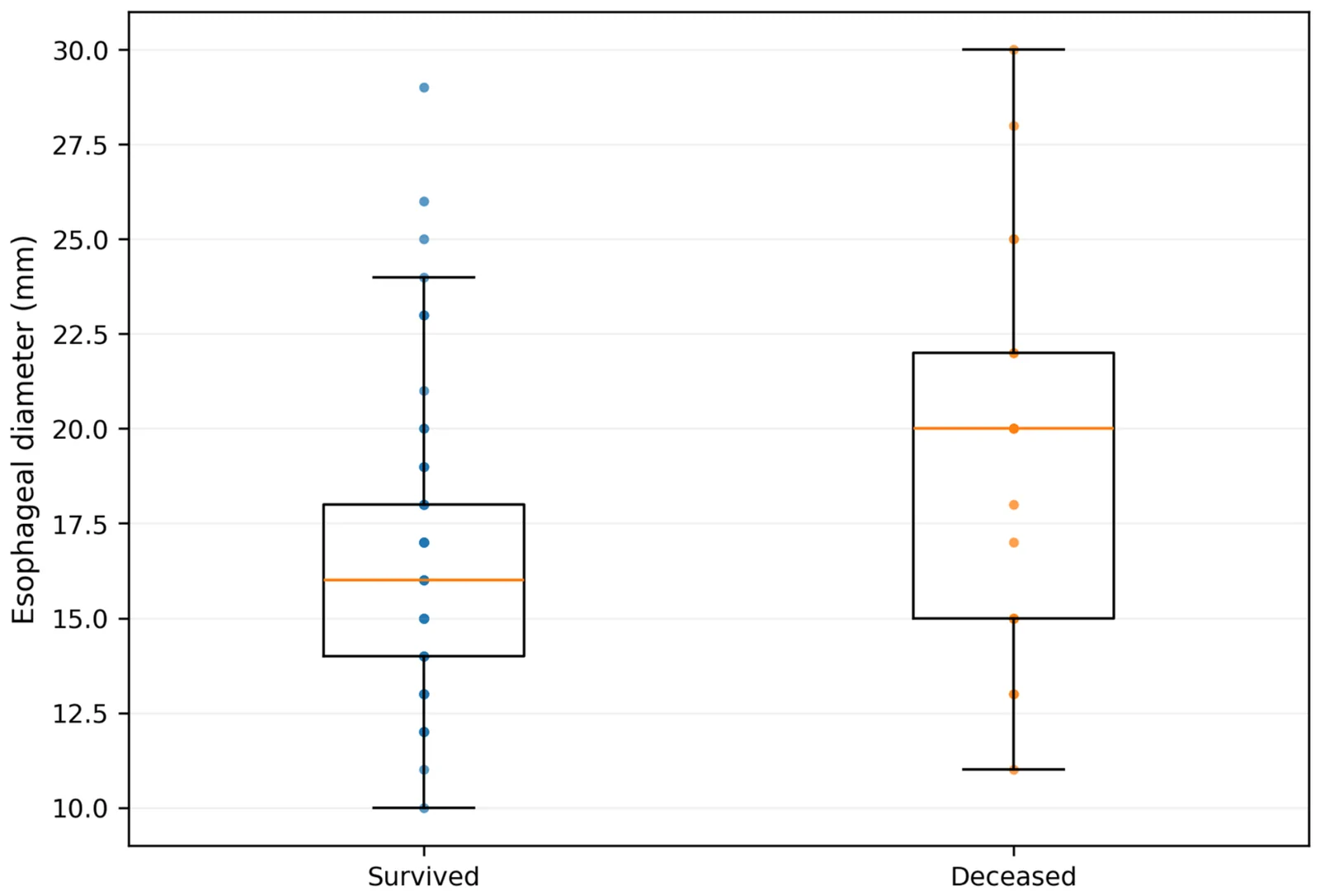
Boxplot representing the esophageal diameter (mm) in the AEW group. Note the smaller esophageal diameter in surviving (left, blue color) vs. deceased (right, orange color) patients; *p* < 0.05. Individual values are represented as circles; medians are represented as horizontal lines.

The mean maximal diameter of the esophagus in deceased patients presenting with AEW was found at 19.2 ± 5.3 (11–28) mm; in the surviving patients, it was found at 16.4 ± 3.7 (11–29) mm (Figure 3). The difference was found at 2.8 ± 0.4 mm and was statistically significant (*p* < 0.05).

Further analysis revealed that cervical AEW of 18.5 ± 4.2 mm was present in 12/19 (63.2%) of the deceased patients, whereas of the surviving patients, 22/79 (27.8%) exhibited AEW in the cervical region, this difference was found to be statistically significant (*p* < 0.05). There was no statistically significant difference in esophageal width among the other three esophageal segments between the groups. *n* = 10 out of the *n* = 19 deceased CIPs were intubated for mechanical ventilation. In an additional analysis, *n* = 6 patients among both intubated and non-intubated deceased CIPs presented with almost similar intragastric air-fluid levels. *n* = 2 deceased patients received prior imaging at 37 days and 13 months before their admission to the ED. Both patients exhibited abnormal esophageal diameters of 18 mm and 13 mm. On prior imaging, no evident widening of the esophagus lumen in the correlating segments could be detected. The other *n* = 17 deceased patients did not receive prior imaging.

When the analysis was repeated with exclusion of intubated/resuscitated patients (*n* = 63 survivors, *n* = 9 deceased), the observed higher esophageal widening in deceased patients remained statistically significant using the pooled *t*-test (*p* < 0.05).

### 3.3. Part 2: Correlation of Serum Biomarkers with Mortality and Exploratory Logistic Regression Analysis for Prediction of Death in AEW Patients

We correlated pathological elevation/lowering of multiple laboratory biomarkers with 28-day mortality in the group of all critically ill patients presenting with esophageal widening using Fisher’s exact test. Statistically significant correlations were found for serum Potassium (*p* < 0.05), serum bilirubin (*p* < 0.05), alkaline phosphatase (*p* < 0.05), lactate (*p* < 0.05), creatinine (*p* < 0.05), NT-proBNP (*p* < 0.05), Leukocyte count (*p* < 0.05), D-Dimer (*p* < 0.05), and hemoglobin (*p* < 0.05).

Non-significant correlations were found for mortality with serum sodium, glucose, gamma GT, Alanine Aminotransferase, C-reactive protein, Procalcitonin, Troponin I, Albumin and for Thrombocyte count.

When the analysis was repeated with exclusion of intubated/resuscitated patients (*n* = 63 survivors, *n* = 9 deceased), statistically significant (*p* < 0.05) correlations were found for Serum Potassium, bilirubin, lactate, Leucocyte count.

#### Stepwise Logistic Regression Analysis

**Figure 4:**
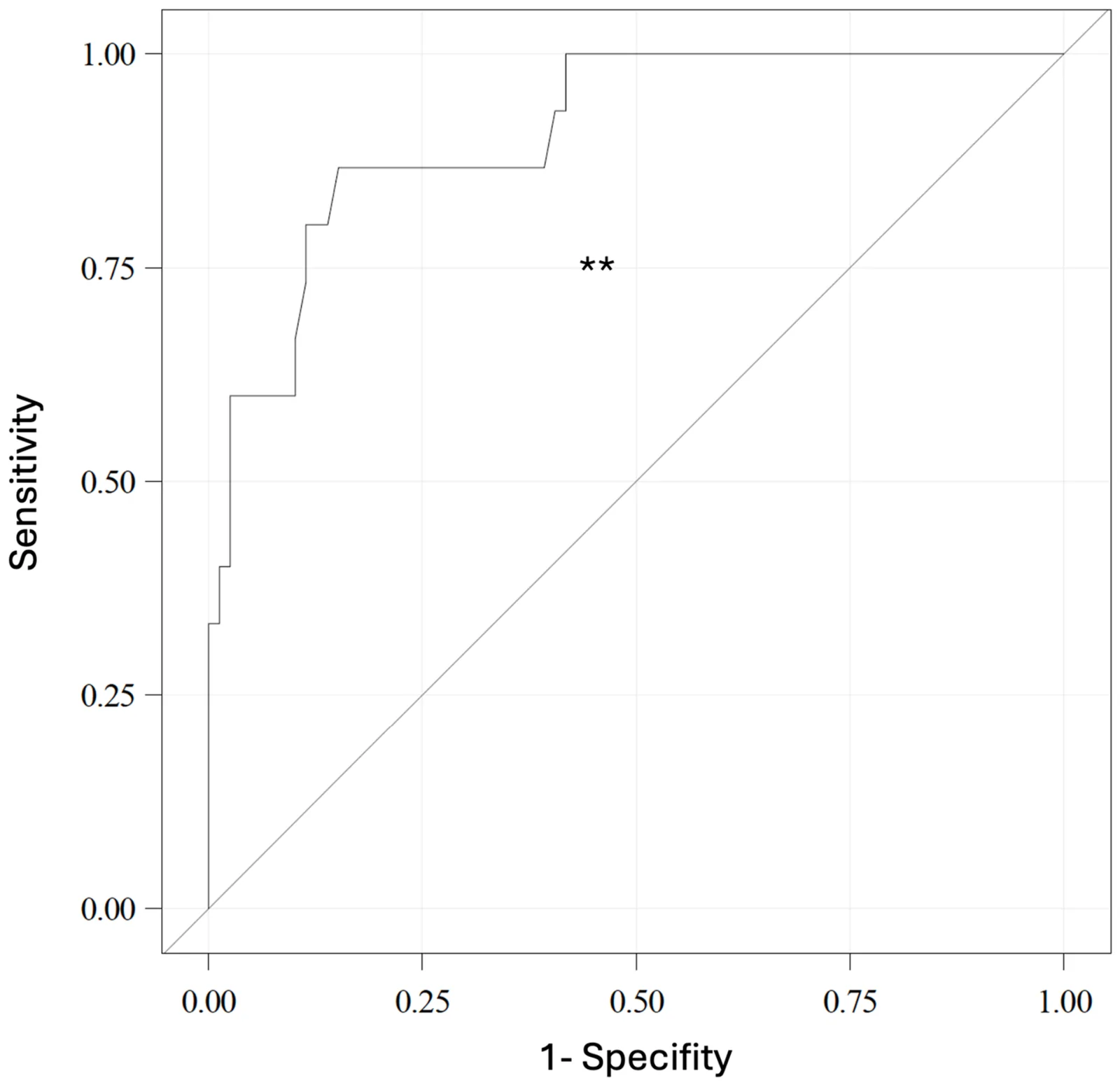
Receiver operating characteristic (ROC) curve demonstrating the performance of a classification model for prediction of death in patients with AEW. Elevated total bilirubin, age, leukocyte count, and hemoglobin were retained in the final model. Cervical AEW as a candidate variable was not retained. The analysis results in an area under the curve (AUC) of 0.9084 (**) for prediction of mortality.

A stepwise binary logistic regression analysis was performed to identify variables associated with mortality (Table 3). Of *n* = 98 patients, *n* = 94 were included in the final analysis, comprising *n* = 15 deaths and *n* = 79 survivors; 4 patients were excluded because of missing values. The stepwise selection retained total bilirubin, patient age, leukocyte count, and hemoglobin in the final model; cervical AEW as a candidate variable was not retained. Increasing age was associated with higher odds of death (OR 1.074 per year, 95% CI 1.014–1.137; *p* = 0.0144). Elevated bilirubin (*p* = 0.0025), leukocyte count (*p* = 0.0040), and lowered hemoglobin (*p* = 0.0292) were also significantly associated with mortality. The final model was found statistically significant (likelihood-ratio χ^2^ = 35.28, *df* = 4; *p* < 0.0001) and showed good discriminatory performance, with an area under the ROC curve of 0.908 (Figure 4).

The sensitivity cohort comprised *n* = 72 patients (*n* = 9 who died and *n* = 63 survivors). For the regression analysis, 3 additional patients were excluded because of missing covariate data, leaving *n* = 69 complete cases, including *n* = 6 deaths. Owing to the limited included patient cohort, the analysis could not be completed. In an additionally performed highly exploratory approach, a backward elimination logistic regression analysis was utilized, which yielded solely elevated serum lactate as a predictor of death (OR 9.52, 95% CI 1.56–58.82, *p* = 0.0147, AUC 0.7024). See the Supplementary Materials for more details.

### 3.4. Part 3: Correlation of AEW Alone with Mortality in All Critically Ill Patients

The correlation between presence of AEW with mortality in all critically ill patients with and without AEW was analyzed for both the whole group of patients and subgroups of patients with different age (Table 4). Overall, no statistically significant differences were found (*p* = 0.776).

## 4. Discussion

This study confirmed the presence of AEW in a significant proportion of all severely ill patients presenting to our ED. Exploratory analyses suggest that a clinical score which includes the presence of AEW besides traditional laboratory-testing derived parameters might assist in patient stratification in the future. The analysis included a broad spectrum of cases from a tertiary care hospital ED utilizing CT for a high proportion of critically ill patients. To our knowledge it was the first study to report insights on this specific CT-based imaging feature.

Esophageal dilation was observed during clinical patient management. In the process of further standardizing and exploring this feature, it became apparent that there is a relative lack of studies that report on esophageal diameters. Schraufnagel et al. reported the length of esophageal lumina that contained air and defined respective cut-offs and did not just measure luminal diameters [14]. Based on the reported results of 92.1% of all measurements presenting with lumina up to 10 mm in diameter, the cut-off of 10 mm was chosen for inclusion into this analysis. Given this background, it cannot be interpreted as a universally accepted threshold. Most of the deceased CIPs in this study suffered from severe illness or injury, which may directly or indirectly affect the CNS. Severe CNS injury has been observed to lead to a loss-of-control for both somatic and smooth muscle cells. AEW in this study was found to correlate with mortality if found in the upper esophageal segment. Whereas the underlying pathophysiological mechanisms have not been explored in detail in this study, the observation of differences within the esophagus is significant. Sarcopenia and mitochondrial dysfunction or chronic inflammation have previously been outlined as relevant factors [18], which may contribute to impairment of esophageal muscle function. For severe COVID-19 infections, a systematic review including 22 studies demonstrated an association between the severity of sarcopenia and mortality in 17/22 studies (77.3%) [19]. Additionally, Bai et al. revealed the combined presence of frailty and sarcopenia a risk factor for 30-day mortality in 653 elderly CIPs [20]. These findings correlate well with the results published by Kahn et al., which demonstrated advanced sarcopenia in patients suffering from liver cirrhosis to increase the risk of sepsis 1.7-fold, leading to 4-times increased mortality risk [21]. The assessment of sarcopenia in CT imaging currently is typically performed by calculating the skeletal muscle index (SMI) at lumbar vertebrae [21,22]. Further histopathological, electrophysiological and electron microscopic assessment would be valuable to elaborate the reasons of AEW in CIP pre- and postmortem as it has previously been performed for both skeletal muscles [23] and gut muscles [24], as also described by Granic et al. [25]. Other physiological factors often present in critically ill patients need to be considered as potential confounders, many of them iatrogenic. Pre-CT mechanical ventilation was found in 10/19 patients who died and presented with AEW. As ventilation may affect the esophageal diameter, the reported results must be interpreted with caution. Accordingly, sensitivity analyses were included to reduce the risk of overstating the results. In cohorts of non-intubated intensive care patients, dysphagia has been reported prevalent in 7.9% of patients on the day of the study examination [26]. Additionally, pharmacotherapy may cause dysphagia and contribute to the presence of AEW. Feeding tubes furthermore represent a mechanical iatrogenic contributor to AEW, whereas AEW has been attributed to natural aging. The age of deceased CIP within this study ranged from 46 to 93 years. Comparably young patients without prior neuromuscular disorder exhibiting AEW support our hypothesis that it may be seen as a separate factor, secondary to severe illness. On the backdrop of these factors, further studies unraveling the potential multifactorial pathogenesis of AEW are necessary.

Significantly increased levels of serum creatinine and bilirubin correlated with mortality in the AEW subgroup. The prognostic value of these serum parameters has been well documented. A retrospective study reported that CIPs with spontaneous bacterial peritonitis were more likely to die if they presented with serum bilirubin levels > 4 mg/dL and serum creatinine levels > 1 mg/dL at the time of diagnosis [27]. Another prospective clinical study demonstrated that CIP with decompensated liver cirrhosis associated with acute renal failure characterized by elevated serum bilirubin and creatinine are more likely to die within 30 days [28]. Another clinical study demonstrated that the combined serum creatinine and bilirubin assay may serve as a biomarker for independently predicting long-term death in patients with ischemic heart disease [29]. These scores typically consider a multitude of parameters like age, total bilirubin, creatinine and INR [7,30,31,32]. This study confirmed that in patients presenting with AEW, serum parameters may help predicting mortality risk. The simultaneously observed correlation of AEW extent with mortality risk suggests, that further evaluation of the parameter to be a promising goal. AEW alone did not correlate significantly with short-term mortality when looking at the larger group of all CIPs receiving CT in this study. Statistical power of this study was limited as the case groups were relatively small. In conclusion, these findings suggest to instead evaluate AEW as part of a refined clinical scoring system, given its availability for performing CT in ED patients, which is becoming a routine procedure, and its measurement is comparably simple.

This study is limited by multiple factors. The study design is monocentric and retrospective, and it has been performed within a limited period of time. Selection bias is therefore inevitable; however, the ED is run by a large tertiary care hospital, ensuring a broad variety of individual morphologies and underlying conditions and diseases. Based on the initial findings reported here, a larger prospective evaluation of AEW is necessary. The lack of published reports on esophagus diameter measurements created the challenge of defining a threshold esophageal diameter for study inclusion which needed to be based on a single published report. The comparably low number of CIPs who died (*n* = 19) compared to the total number of patients included in the study overall limits the power of the statistical analyses, which applied especially to the logistic regression analysis and mortality comparison between AEW/non-AEW patients. The stepwise logistic regression model included *n* = 15 deceased patients and four predictors, which limits the reliability of its predictions. The results need to be interpreted with caution. Another possible limitation on the patient level is that *n* = 10 out of the *n* = 19 deceased CIPs were intubated for mechanical ventilation. Frequently, prior to intubation, manual external ventilation is clinically performed, which makes iatrogenic esophageal dilation a plausible source of bias. After exclusion of patients who underwent intubation or resuscitation before CT, the association between esophageal diameter and mortality remained statistically significant. Given especially the limited case number of only *n* = 6 patients who died within the sensitivity cohort, cautious interpretation of the results is necessary. Although the performed sub-analysis including six patients in both intubated and the non-intubated deceased CIP demonstrate that these patients show almost similar intragastric air-fluid levels, it cannot causally rule out potential bias of treatment affecting the esophageal width. Another important limitation consists of the inability to retrospectively extract comparable sickness severity reports as for example deriving from APACHE II scores. During the very early phase of patient management in our ED, standardized scoring was not performed in all patients, and incomplete documentation did not allow for reliable retrospective assignment of such scores. A future study should be conceptualized to link AEW extent to disease severity, as fluctuations in both the patient’s condition and esophageal calibers have not been explored in detail, and it might be an interesting approach to link AEW measurements to the precise clinical state of the patient at the moment of image acquisition.

## 5. Conclusions

Esophageal widening of the cervical part of the esophagus was found for a substantial amount of critically ill patients. In patients with widened esophagus, greater esophageal diameter was associated with increased 28-day mortality. The reported effects support conceptualization of future studies that could prospectively evaluate CT-reported AEW and its potential future role within multimodal risk assessment of critically ill patients.

## Figures and Tables

**Table 1:** Overview of all (*n* = 1683) included CIP patient examinations who were examined in the ED via CT.

Final Diagnosis	Survived	Death Within 28 Days
Normal esophagus lumen	*n* = 1256	*n* = 329
Dilated esophagus lumen	*n* = 79 (16 intubated)	*n* = 19 (10 intubated)

**Table 2:** List of final diagnosis for the deceased CIPs who expressed esophageal widening. CNS = Central Nervous System.

Final Diagnosis	Number of CIP	Potential Effect on CNS
Intracranial hemorrhage	7	direct
Cerebral infarction	3	direct
Shock		
(cardiogenic, septic or traumatic)	4	indirect
Vascular		

**Table 3:** Multivariable stepwise logistic regression analysis for mortality in the AEW patients. The four displayed parameters were most associated with the risk for death. The presence of cervical AEW was not retained due to its smaller relative contribution to mortality prediction.

Parameter	Odds Ratio	95% Confidence Interval		*p*-Value
Age, per year	1.074	1.014	1.137	0.0144
Elevated total bilirubin vs. normal	76.92	4.63	>1000	0.0025
Elevated Leukocyte count vs. normal	12.20	2.23	66.67	0.0040
Lowered hemoglobin vs. normal	6.06	1.20	30.30	0.0292

**Table 4:** Analysis of 28-day mortality in the group of all included study patients. There was no statistically significant correlation between AEW presence and mortality risk. Note: *n* = 4 cases needed to be excluded from statistical analysis due to incomplete data.

Subgroup	28-Day Mortality (AEW Present)	28-Day Mortality (No AEW Present)	Relative Risk (95% CI)	*p*-Value and Test Utilized
All examinations	19/97 (19.6%)	329/1582 (20.8%)	0.94 (0.62–1.43)	0.776 (Chi-Square test)
<50 years	1/22 (4.5%)	19/499(3.8%)	1.19 (0.17–8.52)	0.585 (Fisher’s exact test)
50–65 years	1/21 (4.8%)	59/412 (14.3%)	0.33 (0.05–2.28)	0.335 (Fisher’s exact test)
>65 years	17/54 (31.5%)	251/671 (37.4%)	0.84 (0.56–1.26)	0.386 (Chi-Square test)

## Data Availability

Study data will be available from the corresponding author upon reasonable request.

## Supplementary Materials

The following supporting information can be downloaded at: https://www.mdpi.com/article/10.3390/jcm15218638/s1. Performed sub-analyses from patients that have not been intubated (demographic analysis, esophageal diameter and laboratory parameters, regression analysis) are included.

## Abbreviations

The following abbreviations are used in this manuscript:

ABIC	Age–bilirubin–international normalized ratio (INR)–creatinine
AEW	Abnormal esophageal widening
AUC	Area under the curve
CIP(s)	Critically ill patient(s)
COVID-19	Coronavirus disease 2019
CT	Computed tomography
ED	Emergency department
kVp	Kilovoltage peak
L3	3rd lumbar vertebra
LIS	Laboratory information system
mAs	Milliampere-seconds
MDCT	Multidetector CT
MEN II	Multiple endocrine neoplasia type 2
RIS	Radiology information system
ROC	Receiver operating characteristics
SD	Standard deviation
SMI	Skeletal muscle index
