# Supplementary material for: Exploring Esophageal Widening in Computed Tomography Performed in Critically Ill Patients
Source: J Clin Med. 2026 Nov 6;15(21):8638. doi: 10.3390/jcm15218638 (PMC13763644; doi:10.3390/jcm15218638)
Supplement: Supplementary file 1 [file jcm-15-08638-s001.zip › jcm-4558051-supplementary.pdf]

Exploring Esophageal Widening in Computed Tomography Performed in Critically Ill Patients

- supplementary materials -

Sub-Analysis of non-intubated patients: Esophageal diameter, patient age

After exclusion of patients who underwent intubation before CT, n=72 patients with AEW remained for analysis, including n=9 patients who died within 28 days and n=63 survivors. Deceased patients were significantly older than survivors (77.4 ± 14.8 vs. 59.7 ± 20.3 years; p = 0.0136). The mean maximal esophageal diameter was greater in deceased patients than in survivors (1.96 ± 0.53 vs. 1.64 ± 0.37 cm; p = 0.0281).

Sub-Analysis of non-intubated patients: Laboratory parameters

In exploratory analyses of laboratory parameters, significant associations with 28-day mortality were observed for abnormal potassium (Fisher's exact p = 0.0375), elevated total bilirubin (p = 0.0141), elevated lactate (p = 0.0030), and leukocyte count (exact p = 0.0026). No statistically significant associations were observed for the remaining evaluated laboratory parameters.

Backward elimination regression analysis

N=69 of the n=72 patients were available as complete cases; three patients were excluded because of missing covariate data. The regression population comprised n=6 deaths and n=63 survivors. The initial full backward logistic regression model showed complete separation, indicating unstable maximum-likelihood estimation. Following backward elimination, lactate was the only variable retained in the final model (p = 0.0147).

An odds ratio of 0.105 (95% CI 0.017–0.642) for normal versus elevated lactate was found. Expressed as elevated versus normal lactate, this corresponds to an OR of 9.52 (95% CI 1.56–58.82; p = 0.0147). The final model had an AUC of 0.7024.
